# Intellectual and Developmental Disabilities: Male Health

**DOI:** 10.3389/fpubh.2014.00208

**Published:** 2014-10-24

**Authors:** Joav Merrick, Mohammed Morad, Eli Carmeli

**Affiliations:** ^1^Health Services, Division for Intellectual and Developmental Disabilities, Ministry of Social Affairs and Social Services, Jerusalem, Israel; ^2^Clalit Health Services, Yaski Community Medical Center, Ben Gurion University of the Negev, Beer-Sheva, Israel; ^3^Department of Physical Therapy, Faculty of Social Welfare and Health Sciences, Haifa University, Haifa, Israel

**Keywords:** intellectual disability, developmental disabilities, male health, sexual dysfunction, physiological, health screening

## Introduction

People with intellectual and developmental disabilities (IDD) are today living and surviving longer and today quite a number also reaching old age. In the past few decades, there has been a marked interest in men’s health, their health promotion, and gender relations has been observed in the general population (1) and recent research about male perspectives has also emerged in the population of people with IDD (2–4).

Despite increased professional and academic interest in men’s health in the general population, a change in government policy to focus on male health issues has been lacking (5). Sexual dysfunction, erectile dysfunction, hypogonadism, and premature ejaculation has been shown to be associated with physical and psychological health, and there is a strong correlation between sexual dysfunction and cardiovascular disease, metabolic syndrome, quality of life, and depression (6). Men also seem to be at greater risk of both developing and dying from those cancers that should affect men and women equally. In the United Kingdom, men have a 69% higher mortality rate and a 62% higher incidence rate for the major cancers, which should affect men and women equally (7). The rate of premature death is correspondingly high, with more than 37,000 additional years of life lost for working-age men (15–64 years) as a result of death from cancer (7).

## Health Screening for Men

The National Institutes of Health and the National Library of Medicine in the United States have made recommendations for males aged 18–39 years (8), 40–64 years (9), and 65 years and older (10), which can readily be adapted for males with IDD and used for health encounters with the purpose of:

- Screening for possible diseases and disorders
- Assessing the risk for future medical problems
- Helping develop a healthy lifestyle
- Updating vaccinations and
- Maintain a good relationship with the physician

Many health problems are silent, and the person may not know, for example, if he has high blood pressure unless it is measured or the consequences of high blood pressure become apparent. The following health screening is therefore recommended for males with IDD (8–12):

- Blood pressure screening on a yearly basis for adults over 18 years of age
- Cholesterol/lipid/glucose screening and heart disease prevention yearly from age 35 years
- Dental exam every year for an exam and cleaning
- Vision and hearing exam yearly
- Immunizations: after age 19 years, a tetanus–diphtheria and acellular pertussis (Tdap) vaccine once as part of tetanus–diphtheria vaccines and tetanus–diphtheria booster every 10 years. HPV vaccine should be considered. Shingles or herpes zoster vaccination once after age 60 years and also pneumococcal vaccine. The flu vaccine every year.
- Infectious disease screening: depending upon lifestyle and risk behavior screening for infections such as syphilis, chlamydia, and HIV, as well as other infections.
- Checking height and weight/obesity/body mass index yearly
- Screening for alcohol and tobacco use and counseling
- Screening for depression/mental health regularly
- Colon cancer screening with fecal occult blood yearly after age 50 years. Sigmoidoscopy and colonoscopy if possible every 5–10 years, but more often if a personal or family history
- Osteoporosis screening with yearly screening at age 40 years for people in residential care and age 45 years for community residents
- Prostate cancer screening with PSA blood test should be considered after age 50 years
- Abdominal aortic aneurysm screening at age 65–75 years by abdominal ultrasound examination

## Male Health and Intellectual Disability

There are differences in male health for people with IDD compared to the general population and compared to women (13). Males with IDD have less risk taking behavior, will usually have more regular preventive health care, and persons with severe and profound IDD are less likely to smoke or drink alcohol due to their functional limitations (13).

### Life expectancy, mortality, suicide, and cancer

A 35-year follow-up study from Finland based on a nation-wide population study of people with IDD has shown that males die younger than females (14) and the three most common causes of death were cardiovascular diseases, respiratory diseases, and neoplasms (15). Disease mortality was high up to 40 years of age, but did not increase thereafter, and the difference among gender in cause-specific mortality was smaller than in the general population (15).

Suicide mortality was significantly low among males with IDD (16), at only one-third of the general population risk, while females with IDD are at equal suicide risk to Finnish women in general. Risk factors for suicide were similar to those in the general population. Most suicide victims with IDD had mild IDD and comorbid mental disorders. Suicide methods were passive, and alcohol was involved in only one case.

In another study from the same Finnish group (17), the risk of neoplasms among people with IDD was studied. The observed number of cancers in the cohort was close to what was expected and there was a significantly reduced risk of cancers of the prostate, urinary tract, and lungs. The risk was increased in cancers of the gallbladder and thyroid gland. The risks of lung and gallbladder cancer were lowest and highest, respectively, in people with profound and severe IDD, a group who also had significantly elevated risk for brain cancer and testicular cancer. The incidence of cancer among people with IDD was comparable with the general population.

The same group also looked at people with Down syndrome (DS) and fragile X. For people with fragile X, there was no statistically significant difference with the general Finnish population, but an increased risk for lip cancer was found (18). Individuals with DS were found to have an overall equal cancer risk to that of the general population, but a significantly higher risk of leukemia and testicular cancer (19). This higher risk for leukemia in DS was also found in an Israeli study (20), which also observed a significant excess of gastric cancer in males with DS.

Based upon the nation-wide Danish registration of people with IDD since 1850s compared to the general population, it was found that for males with IDD aged 15–64 years, there was an excess mortality of malignant neoplasms, diseases of the respiratory system, congenital malformations, and accidents (21).

## General Health

There is very little research or data specifically on the health of males with IDD, which is an interesting observation on its own.

In a research study (22) with 2,282 adults with IDD, aged 40 years and older, it was found that diseases occurred at about the same frequencies for males and females, but age-related changes in most organ-system diseases differed by gender. The prevalence of cardiovascular disease, cancer, as well as impairments of vision and hearing increased with age groupings for both genders. The only disease that did not significantly increase with age grouping in either females or males was hematological disorders. Psychiatric disorders decreased with age in males, while neurological disease decreased with age only in females. In a larger combined study (23) from New York State, Israel (22), and Taiwan with 4,449 adults with IDD, it was found that males were less likely to have endocrine, infectious, and respiratory diseases than females.

In a smaller study (24) with 101 persons from a community sample compared to a 101 sample from residential care of older adults with IDD, no significant effect was found for gender and no significant residence versus gender interaction; but, for each disease separately it was found that males displayed more cardiovascular problems (9%) than females (1%) and females displayed more visual problems (37%) than males (21%).

## Specific Health Issues

There are some health issues specific to males with IDD, but it should be noted that symptoms and presentations are not always as clear or evident as for a male in the general population. One example from our work is the case of Alex.

Alex, a 45-year old male with mild IDD living in a small group home and working in a regular job wanted to get himself a new pair of pants. He went to his regular clothes store to see what he could find. Alex found some pants that he liked and went to the dressing room to try them on out. It took him longer time than usual to handle this task, so the sales person, who knew Alex from earlier encounters asked Alex if he needed help. Alex said yes and when the sales person saw Alex in his underwear, he noted a large bulge on his left side of his scrotum and realized that this was part of the reason why Alex was having difficulty trying on pants – they were too tight. The sales person, who knew of the group home where Alex lived, asked Alex if he could talk to the staff there and tell them about his problem. Alex gave his permission and as a result Alex was referred for a medical examination and a testicular tumor was found and treated.

### Androgen deficiency

Androgen deficiency is more common in males with IDD than in general population (13) and, in early teenage years, may result in failure of testis and penis to grow, failure of facial, body and pubic hair to appear, failure of voice to deepen, and failure of muscle development and growth (13). In adult untreated males, it can result in decreased energy, altered mood, irritability, impotence, infertility, hot flushes, gynecomastia, and osteoporosis (13).

Hypogonadism is more prevalent in males, for example, with DS, Klinefelter and Prader–Willi syndrome and the diagnosis is made by measurement of serum testosterone and luteinizing hormone levels. Testosterone treatment is today available.

### Cryptoorchidism

Common in males with DS, Prader–Will, and Noonan syndrome and intra-abdominal testis is a risk for testicular cancer (13). Surgical treatment is available.

### Prostate

In a study (25) of 18 Hong Kong residential care facilities (811 adults with IDD) compared to the general population, it was found that adult males with DS showed higher prevalence of prostatic hypertrophy (0.9 versus 0.3%).

In an Australian study (26) of 9,409 persons with IDD, males were observed to have a significantly increased risk of leukemia, brain and stomach cancers, and a reduced risk of prostate cancer.

### Sterilization

Forced sterilization or sterilization without consent has been part of disability history, and it is now performed on a much smaller scale than in the past, but still taking place although now only in, for example, the UK through a court decision (27). Guidelines for this procedure in children and youth have been published by the American Academy of Pediatrics (28).

Health is like money, we never have a true idea of its value until we lose it.

Josh Billings (1815–1885) American humorist and lecturer

## Conclusion

The story of Alex above teaches us the importance of regular medical/health check-ups and screening with full examination including genitalia, even in the case of persons who have mild IDD, living at home or in group home and look healthy and carry on a normal job and life. The same though holds true for the general population in fact, since usually a person visiting the general practitioner for a sore throat will not, for example, have his genitalia checked. Primary health care for this population is important and we need to find ways to provide a golden standard of care and service that cater to this population (11).

Recently, the Vanderbilt Kennedy Center for Excellence in Developmental Disabilities, together with Tennessee’s other university Center of Excellence in Developmental Disabilities, the Boling Center, and the Tennessee Department of Intellectual and Developmental Disabilities have developed an e-toolkit using experiences from Canada, which also has a checklist for male health (29).

The paucity of research in the literature on health and medical issues that are particular to men with IDD is of some concern. While the studies referenced in this paper are beginning steps toward advancing our knowledge of male-specific manifestations of disease and its treatment, more detailed research on the male population with IDD is needed. Furthermore, the field of healthcare and IDD will benefit from the study of health promotion and disease prevention that may be particular to men. We therefore recommend a research agenda that focuses on males looking at the health status, healthcare needs, health promotion, and disease prevention, including diet, physical activity, and exercise needs of men with IDD be developed as a matter of utmost priority.

## Conflict of Interest Statement

The authors declare that the research was conducted in the absence of any commercial or financial relationships that could be construed as a potential conflict of interest.
